# Group mindfulness based cognitive therapy vs group support for self-injury among young people: study protocol for a randomised controlled trial

**DOI:** 10.1186/s12888-015-0527-5

**Published:** 2015-07-08

**Authors:** Clare S. Rees, Penelope Hasking, Lauren J. Breen, Ottmar V. Lipp, Cyril Mamotte

**Affiliations:** School of Psychology and Speech Pathology, Curtin University, GPO Box U1987, Perth, WA 6845 Australia; School of Biomedical Science, Curtin University, Perth, WA 6845 Australia

**Keywords:** Non-suicidal self-injury, Mindfulness based cognitive therapy, Randomised controlled trial

## Abstract

**Background:**

Non-suicidal self-injury (NSSI) is a transdiagnostic behaviour that can be difficult to treat; to date no evidence based treatment for NSSI exists. Mindfulness Based Cognitive Therapy (MBCT) specifically targets the mechanisms thought to initiate and maintain NSSI, and thus appears a viable treatment option. The aims of the current study are to test the ability of MBCT to reduce the frequency and medical severity of NSSI, and explore the mechanisms by which MBCT exerts its effect.

**Methods/Design:**

We will conduct a parallel group randomised controlled trial of Mindfulness Based Cognitive Therapy (MBCT) versus Supportive Therapy (ST) in young people aged 18–25 years. Computerised block randomisation will be used to allocate participants to groups. All participants will meet the proposed DSM-5 criteria for NSSI (i.e. five episodes in the last twelve months). Participants will be excluded if they: 1) are currently receiving psychological treatment, 2) have attempted suicide in the previous 12 months, 3) exhibit acute psychosis, 4) have a diagnosis of borderline personality disorder, or 5) have prior experience of MBCT. Our primary outcome is the frequency and medical severity of NSSI. As secondary outcomes we will assess changes in rumination, mindfulness, emotion regulation, distress tolerance, stress, and attentional bias, and test these as mechanisms of change.

**Discussion:**

This is the first randomised controlled trial to test the efficacy of MBCT in reducing NSSI. Evidence of the efficacy of MBCT for self-injury will allow provision of a brief intervention for self-injury that can be implemented as a stand-alone treatment or integrated with existing treatments for psychiatric disorders.

**Trial registration:**

Australian New Zealand Clinical Trials Registry Number ACTRN12615000023550. Registered 16 January 2015.

## Background

Non-suicidal self-injury (NSSI), the deliberate destruction or alteration of body tissue without conscious suicidal intent and for purposes not socially sanctioned [1], is a transdiagnostic behaviour that is used to cope with intense emotions and psychological distress. Equally common among males and females, NSSI can include cutting, burning or carving the skin and hitting or banging the self or hard objects. Although typically emerging in adolescence, NSSI is most prevalent among 18–24 year olds (20 % lifetime history) [2]. NSSI differs from suicidal behaviour (including ideation and attempts) in being more prevalent, engaged in more frequently, typically involving non-lethal methods, and being driven by emotion regulation rather than a desire to end life. As such the aetiology of NSSI is markedly distinct from suicidal behaviour, necessitating a tailored treatment approach [3, 4].

In Australia, the direct hospital costs of non-suicidal self-injury are estimated at over $14 million per month [2]. Among 15–24 year olds, 7301 were hospitalised for self-harm (including suicidal behaviour) in 2009–2010, placing the direct cost in this age group alone at almost $34 million per year [5]. With increased awareness and efforts to improve help-seeking [6], these costs are set to rise dramatically unless effective interventions to minimise the frequency and medical severity of the behaviour are made available. Young people who self-injure carry a fourfold risk of suicidal thoughts and behaviours within the following year [7], and Australians who self-injure are 42 times more likely to attempt suicide [2]. Reducing the major risk factor of self-injury is crucial to suicide prevention efforts yet there is no targeted evidence-based treatment for self-injury.

Growing recognition of the prevalence and impact of NSSI has led to its inclusion in Section 3 of the latest edition of the *Diagnostic and Statistical Manual of Mental Disorders* (DSM) [8], as a condition for further study. Proposed criteria include: NSSI on five or more days in the past year, for the purpose of relief from negative feelings, resolving interpersonal difficulties or to induce a positive state (e.g. euphoria). NSSI is defined as being associated with negative thoughts and feelings, premeditation and rumination. The proposed criteria evidence discriminant validity [9–11].

Commensurate with the affect-regulatory function of NSSI, mechanisms thought to increase risk of NSSI in the face of stress include poor emotion regulation, poor distress tolerance and rumination on negative thoughts and feelings. Emotional cascade theory proposes that through rumination even minute emotional stimuli become amplified over time [12] and, in the absence of adaptive emotion regulatory strategies, individuals self-injure to escape the subsequent cascades of intense emotion. Supporting this, researchers consistently demonstrate increased arousal and rumination prior to NSSI [13, 14], higher physiological reactivity and poor distress tolerance when under stress [15], and emotional relief and reduced arousal after NSSI [16].

### Treatment for NSSI

Despite the adverse outcomes for people who engage in NSSI, there is no empirically validated treatment available [17]. Where NSSI is an outcome variable of interest in clinical trials, treatment has been exclusively designed for people with Borderline Personality Disorder (BPD) [18–20]. Given the transdiagnostic nature of NSSI, treatments that address NSSI beyond BPD are essential. Mindfulness-Based Cognitive Therapy (MBCT) was designed specifically to impact on attention to negative thoughts and images, emotion regulation, distress tolerance and rumination [21, 22], and successfully evidences reductions in emotional reactivity [23]. That this treatment directly addresses proposed diagnostic features of NSSI, and core mechanisms repeatedly implicated in NSSI [8], offers immense promise for MBCT as a treatment for NSSI. Yet, despite recent work implicating a lack of mindfulness in the maintenance of NSSI [24], no prior research has explored the ability of MBCT to impact the frequency and medical severity of NSSI. Garnering support for MBCT as a viable and efficacious treatment is essential to the on-going effort to reduce the impact and burden of NSSI.

If, as theorised, MBCT exerts its effect by minimising attention on negative thoughts and rumination, improving distress tolerance and emotion regulation, and reducing perceived and physiological stress responses, changes in these variables should be evident upon completion of an MBCT program, with subsequent improvement in NSSI. Assessing mechanisms of change is crucial to determining the ‘active ingredient’ in MBCT, facilitating optimal outcomes and tailored treatment approaches [25].

### The current study

**Fig. 1:**
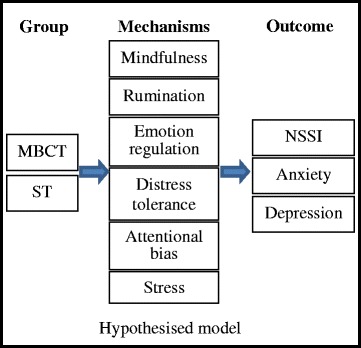
Hypothesised model

In this study we aim to: 1) determine the efficacy of group Mindfulness Based Cognitive Therapy (MBCT) in decreasing the frequency and medical severity of Non-Suicidal Self-Injury (NSSI) among young people, relative to group Supportive Therapy (ST); and 2) establish the mechanisms by which MBCT reduces frequency and severity of NSSI. The following hypotheses are proposed:

1. We expect MBCT to reduce the frequency and medical severity of NSSI, relative to ST and this reduction to be maintained at 3 month and 6 month follow-up
2. We expect MBCT to increase mindfulness, reduce rumination, improve emotion regulation, improve distress tolerance, reduce bias for negative stimuli, and reduce stress (self-reported and physiological, based on cortisol)
3. We expect the proposed mechanisms of action will mediate the relationship between assigned treatment group and outcomes of reduced NSSI, lower scores on the Beck Anxiety Inventory, and lower scores on the Beck Depression Inventory-II (Fig. 1).

## Methods/Design

### Study design

**Fig. 2:**
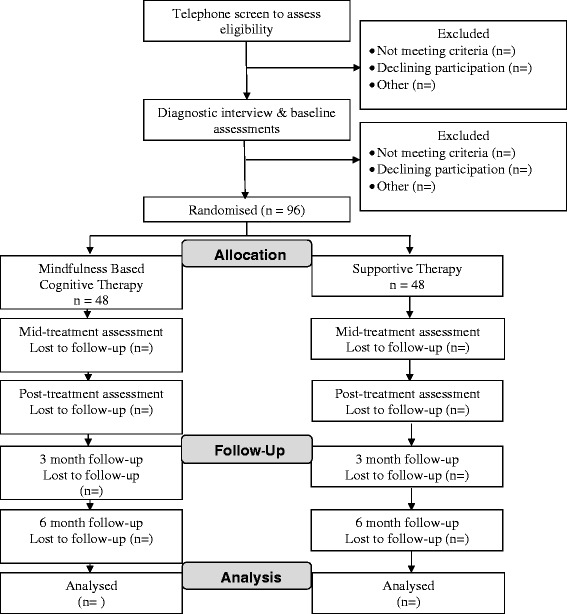
CONSORT flow diagram of participant allocation

We will conduct a parallel group randomised controlled design in which half our participants receive Group Mindfulness Based Cognitive Therapy and half receive Group Supportive Therapy. We will implement a computerised block randomisation procedure to ensure equal distribution of participants across conditions (Fig. 2). As far as practically possible participants will be blind to group allocation. Self-report, behavioural and physiological measures will be administered at pre-treatment, mid-treatment, post-treatment, and 3 and 6-month follow-up. To maximise external validity, a minimum follow-up period of 6 months is recommended and will be adopted in the present study [26]. Baseline assessments and follow up assessments will be conducted by a researcher blind to group allocation.

### Participants and recruitment

We will recruit 96 participants (48 per group) via our existing referral networks of GPs, mental health professionals, and university counselling services, as well as through advertising in print media and social network sites. Inclusion criteria include: 1) Aged 18–25 years, and 2) meet proposed DSM-5 criteria for NSSI [8]. Participants will be excluded if they: 1) are currently receiving psychological treatment, 2) have attempted suicide in the previous 12 months, 3) exhibit acute psychosis, 4) have a diagnosis of borderline personality disorder (BPD), or 5) have prior experience of MBCT. As NSSI is one diagnostic criterion for BPD, young people diagnosed with BPD will be excluded to avoid confounding diagnosis and behaviour. Individuals presenting with suicidal behaviour, psychosis or BPD will be appropriately referred.

### Intervention

A trained psychologist will conduct telephone interviews to assess eligibility with the single-item Clinician-Rated Severity of Non-Suicidal Self-Injury [27] and 6-item Mini International Psychiatric Interview [28]. Both treatment conditions will comprise 8 weekly group sessions of 2 h duration with up to 12 participants in each group. All group sessions and assessments will occur at the Curtin University Psychology Clinic. As per standard practice, two trained therapists, blind to the results of baseline and follow-up assessments, will facilitate each group. Suicidality will be monitored weekly throughout treatment (both conditions) using the 3-item Self-Monitoring Suicide Ideation Scale [29].

To ensure treatment fidelity, each therapist will receive training and weekly supervision. All treatment groups will be audiotaped with 10 % checked to ensure adherence to manualised treatment protocols, using the Mindfulness Based Cognitive Therapy Adherence Scale for MBCT group [30]. At the first treatment session all participants will complete the 6-item Credibility/Expectancy Questionnaire [31] to assess treatment face validity. We will assess treatment acceptability using Dear et al’s [32] 4-item acceptability rating scale. Upon completion of treatment, participants will also provide open comments about acceptability, and specify intended NSSI prevention strategies.

### Mindfulness-based cognitive therapy (MBCT)

We will utilise the standard MBCT treatment protocol [21]. Each session combines key elements of cognitive therapy with training in mindfulness meditation. Participants are taught skills designed to foster present moment awareness which include practising mindfulness meditation, body scan, mindful walking and stretching. Cognitive therapy techniques include education about the role of negative thoughts and how rumination, avoidance, suppression, and struggling with unhelpful cognitions and emotions can perpetuate distress rather than resolve it. Participants learn to identify patterns of emotional response and negative thinking that act as warning signals for NSSI and help one another to develop crisis plans and actions to take in the event of future NSSI urges.

### Supportive therapy (ST)

Supportive therapy is a widely used active control condition in psychotherapy outcome studies, as it controls for both the non-specific effects of any psychological intervention (i.e. therapeutic relationship) and the unique aspects of group therapy (i.e. social support). Additionally, from an ethical perspective, the group support condition ensures that no participants are left without an intervention. We will use Borkovec and Costello’s [33] manualised protocol. In ST, the therapist provides empathy, fosters a supportive environment, and facilitates discussions among group members around NSSI and other life issues. No MBCT techniques are taught by the therapist.

### Ethical issues

After initial telephone screening to assess eligibility, during which participants are verbally informed of the study requirements, all participants will be provided with a detailed information sheet which outlines the aims and participation requirements of the study, informs participants of the confidential and voluntary nature of participation, and outlines how data are to be collected, used and stored in accordance with relevant Privacy Legislation. All participants will be afforded the opportunity to ask questions about the study and will provide signed consent to participate in the research project. Participants are free to withdraw from the research project at any time.

There is some concern regarding the potential for social contagion when discussing NSSI in a group setting. Our experience and a growing evidence base, suggests that when appropriately addressed, iatrogenic effects are rarely observed [34]. Conversely, research indicates that participants benefit from being asked about their NSSI [35, 36]. We will minimise risk of iatrogenic effects by following established guidelines to reduce social contagion [37], including discouraging sharing of explicit details, NSSI images or scars in the group setting. This conduct of this trial has been approved by the Curtin University Human Research Ethics Committee (Ref number: 4884).

#### Clinical outcome measures

##### Self-injury monitoring diary:

Participants will complete the Self-Injury Monitoring Diary, developed for this study, across the course of the trial, to assess ongoing frequency and medical severity of NSSI. Each day participants will indicate whether they had an urge to self-injure, the strength of this urge, whether they did self-injure, and the severity of the injury.

##### Clinician-rated severity of Non-suicidal self-injury

[27]: This single item scale assesses the severity of NSSI on a scale of 0 = None; 1 = Sub-threshold; 2 = Mild; 3 = Moderate; and 4 = Severe. The measure was designed to capture clinically meaningful changes in NSSI severity, based on the proposed DSM-5 criteria [8]. Therapists will rate each participant at the end of each therapeutic session.

##### Beck depression inventory

- 2^nd^ Edition [38] (BDI-II): The BDI-II is a 21-item measures of depressive symptoms, each rated on a four-point scale. Both a continuous measure indicating severity of symptoms, and clinically meaningful cut-off scores can be obtained. The BDI-II is the gold-standard questionnaire assessment of depression and demonstrates acceptability as a screening tool in both healthy and clinical populations [39, 40].

##### Beck anxiety inventory

[41] (BAI): The BAI is a 21-item assessment of anxiety symptoms experienced in the last week, with each symptom rated on a four-point scale. Both cognitive and somatic symptoms are assessed. As the gold standard questionnaire assessment of anxiety, the BAI demonstrates excellent psychometric properties [41].

#### Mechanism of change measures

##### Cognitive and affective mindfulness scale - revised

[42] (CAMS-R): The CAMS-R is a brief (12 item) self-report measure designed to assess the capacity an individual has to be mindful. The measure demonstrates internal consistence and convergent validity with similar measures when administered to university students [42].

##### Ruminative thought style questionnaire

[43] (RTSQ): The RTSQ is a 20-item measure describing positive, negative and neutral facets of global rumination (e.g., “I can’t stop thinking about some things” or “I have never been able to distract myself from unwanted thoughts”). Respondents rate each statement on a 7-point Likert scale (1 = *not at all descriptive of me*, 7 = *describes me very well*). The RTSQ has demonstrated good convergent validity with the Response Style Questionnaire, the Global Rumination Scale and the Beck Depression Inventory, adequate test-retest reliability and high internal consistency [43].

##### Difficulty in emotion regulation scale

[44] (DERS): The DERS uses 36 items to tap into 5 aspects of emotion regulation: non-acceptance of emotional response, difficulty in goal directed behaviour, impulse control, emotional awareness, lack of emotion regulation strategies and emotional clarity. Participants respond to each item on a 5-point scale. The measure demonstrates acceptable reliability in university students as well as content and convergent validity [44].

##### Distress tolerance scale

[45]: This 14 item self-report measure assesses an individual’s ability to withstand feeling distressed. The scale assesses an individual’s ability to tolerate emotions, their appraisal of emotional situations, how absorbed they are by negative emotion and emotion regulation using 5-point Likert scales.

##### Perceived stress scale

[46]: The Perceived Stress Scale is a 10-item assessment designed to provide a global assessment of perceived stress. Items assess how unpredictable, controllable and overloaded individuals find their lives, without reference to specific events. The scale evidences discriminant validity with depression, and internal consistency [46].

##### Cortisol measurement:

Both cortisol awaking response (CAR) and daily slope (DS) will be assessed to obtain total daily cortisol output. Cortisol is a biomarker of stress and anxiety [47], and varies in response to stress among people who self-injure [48, 49]. Following best practice [50] we will collect saliva on two consecutive days at each data collection point. On each of these days, participants will collect saliva upon waking, 30 and 45 min after waking (CAR) and again at 4, 9, and 13 h after waking (DS). To increase adherence to the protocol participants who comply with at least 80 % of saliva collection will receive a $50 iTunes voucher. Participants will use Sarstedt Cortisol Salivettes® to collect saliva, which provides an easy and hygienic collection method, further encouraging compliance. Saliva is obtained by chewing on a synthetic swab which is then placed in the container for safe transportation to the laboratory for analysis. Importantly, the Salivettes® are designed to allow reliable analysis from small saliva volumes and low cortisol levels. Following collection, we will recover the saliva from the Salivettes®, centrifuge and store in aliquots at −80 °C until analysis using an enzyme-linked immunosorbent assay (ELISA). Participants will record waking time and all sampling times, and bring their clearly labelled samples to the subsequent assessment session in the clinic.

##### Attentional bias

The differential allocation of attention to emotional stimuli can be assessed in simple reaction time tasks that are presented using a computer. One of these tasks is the so called ‘dot probe’, which assesses the effect of emotional cues on the detection or identification of a probe stimulus [51]. In this task, two pictures or two words that differ in emotional valence (cues; pleasant and neutral or unpleasant and neutral) are presented simultaneously for a short period of time in different locations on a computer screen, left and right half or upper and lower half. After 500 ms, a probe stimulus is presented in the location that was previously occupied by one of the cues. The probe used in our task are two dots either arranged horizontally ‘**..**’ or vertically ‘**:**’. The participant’s task is to indicate the arrangement of the dots by pressing one of two buttons. Participants will be faster to respond to the probe if their focus of attention is at the location at which the probe is presented. If participants are faster to respond to probes that replace unpleasant cues, we conclude that attention is biased towards the unpleasant stimuli indicating preferential processing. If participants are faster to respond to probes that replace neutral cues, we conclude that attention is biased away from unpleasant stimuli indicating avoidance. Pleasant stimuli are included to assess whether the differential allocation of attention is driven by cue valence (differs for pleasant and unpleasant cues relative to neutral cues) or emotional arousal (is similar for pleasant and unpleasant cues relative to neutral cues).

Cues will be pictures (neutral, pleasant, unpleasant, NSSI related) drawn from the standardised International Affective Picture System [52] and normed words (neutral, pleasant, unpleasant, NSSI related) [53]. Cue location will be counterbalanced (e.g. pleasant cues will appear equally often in each possible location) as will be relation of probe to cue (i.e., the probe will follow an emotional cue equally often than a neutral cue - so cues are not predictive of probe position). This results in 24 different stimulus configurations (trials) for a fully counterbalanced design (3 × 2 × 2 × 2; cue valence [pleasant, unpleasant, NSSI related] x cue location [top vs. bottom/left vs right] x probe location [emotional, neutral] x stimulus material [words, pictures]). These 24 stimulus configurations will be repeated 10 times in a random order yielding a total of 240 trials. Including practise trials, this procedure will take approximately 10 min.

### Sample size calculation and statistical analysis

We will perform both Intention to Treat and Per Protocol analyses. We will conduct a sensitivity analysis [54], and compare complete cases versus cases lost to follow-up on baseline characteristics and scores on clinical measures, by randomization group [55]. Mixed Model Repeated Measures analyses will compare results on outcome measures across conditions and time points, while controlling for random effects (e.g. age, gender). Using medium effect sizes [56] 40 per group are required for power = .80 and α = .05 (G*Power) [57]. Recruiting an additional 20 % (*n* = 48 per group) will allow for typical dropout [58, 59]. Mechanisms of action (see Fig. 1), will be tested using multiple mediational analysis with 1000 bias-corrected bootstrap samples in MPlus, to test total and specific indirect effects. This powerful test of mediation requires a sample of 71 participants to detect a medium effect [60]. Participants will be classified into outcome categories (Recovered, Improved, Unchanged, Deteriorated) [61] according to Reliable Change Index and Clinical Significance of change at post-treatment and follow-up, to determine whether MBCT and ST differ in clinically significant reductions in NSSI and associated symptoms of anxiety and depression over time.

## Discussion

Although delineated from suicidal behaviour by definition, people who repeatedly self-injure carry a fourfold risk of suicidal thoughts and behaviours within the following year [7], and are 42 times more likely to attempt suicide [2]. Of note, NSSI increases risk of suicidal behaviour over and above risk conferred by comorbid psychopathology, adverse life events and psychosocial risk factors [7, 62]. To reduce the physical, psychological, social and economic burden of self-injury, effective interventions are urgently needed [63]. MBCT is a transdiagnostic intervention that has established evidence in the treatment of many clinical conditions. Importantly, MBCT was designed specifically to focus on the very mechanisms thought to maintain self-injury (i.e. rumination, focus on negative thoughts, poor emotion regulation). MBCT thus offers untapped potential for reducing the frequency and medical severity of self-injury.

This trial offers significant advantages over previous efforts to determine effective treatments for NSSI. First, the components of MBCT directly align with cognitive and emotional factors related to NSSI, thus offering the best chance of targeting the key mechanisms maintaining the behaviour. Second, we will collect both self-report and clinician ratings of NSSI severity through the course of the project. These will be supplemented with behavioural measures of attentional bias and analysis of cortisol as a physiological measure of stress. These objective measures significantly strengthen the scientific rigour of the findings. Third, the active comparison condition allows additional control over the influence of group processes, therefore allowing a direct test of the specific intervention mechanism of MBCT.

While the proposed sample size is small, the repeated measures design ensures our study is sufficiently powered to identify medium effects on our primary outcome variable, and provide preliminary assessment of the mechanisms of change. Restricting our sample to 18–25 years necessarily will limit generalisation of the findings to other age groups, however given this is the age at which NSSI is most common [2] it seems the appropriate age range to first test the efficacy of MBCT in reducing this behaviour.

By directly addressing the mechanisms maintaining self-injury irrespective of psychiatric diagnosis, group mindfulness-based cognitive therapy (MBCT) offers great promise as a transdiagnostic treatment that can be a successful intervention for a greater number of young people who self-injure. Establishing efficacy of MBCT for self-injury will provide the first targeted evidence-based treatment, giving therapists the power to intervene confidently and produce positive outcomes for youth who self-injure [64]. Outcomes of this project will significantly improve the care given to those who self-injure, improve their well-being, decrease their chances of further self-injury, and decrease the chance they will die by suicide.

## Abbreviations

CAR: Cortisol awaking response
DS: Daily slope
DSM: Diagnostic and statistical manual of mental disorders
MBCT: Mindfulness based cognitive therapy
NSSI: Non-suicidal self-injury
ST: Supportive therapy
